# Ethnic minority vocal traditions and psychological wellbeing: ethnic-cultural identity and self-reported emotion regulation in Chinese conservatory programs

**DOI:** 10.3389/fpsyg.2026.1981035

**Published:** 2026-10-21

**Authors:** Linhao Qin, Meitian Zhao

**Affiliations:** Department of Music, Taiyuan Normal University, Jinzhong, Shanxi, China

**Keywords:** emotion regulation, ethnic vocal traditions, ethnic-cultural identity, mixed-methods research, music education, psychological wellbeing

## Abstract

**Introduction:**

Ethnic minority vocal traditions may support psychological wellbeing, but comparative evidence remains limited. This study compared Chinese ethnic minority and Western vocal training and examined the associations of emotion regulation, ethnic-cultural identity, training intensity, and instructor–student cultural congruence with wellbeing.

**Methods:**

An observational mixed-methods study included 480 vocal students (240 per tradition) from seven institutions, a longitudinal subsample of 192 students followed for six months, and 40 semi-structured interviews. Quantitative analyses used ANCOVA, regression, parallel and moderated mediation with bootstrapping, and multilevel models. Qualitative data were examined using codebook thematic analysis.

**Results:**

Chinese ethnic vocal training was associated with higher wellbeing (*β* = 0.397), with statistical indirect associations through cognitive reappraisal (0.098) and ethnic-cultural identity (0.139). Ethnic-cultural identity showed the largest between-group difference (*d* = 0.78). The indirect associations remained robust under clusterbased inference, whereas the direct association did not. Training intensity was associated with a stronger emotion-regulation pathway, although this finding was borderline under cluster-based inference. Cultural congruence did not significantly moderate the identity pathway, and no curvilinear moderation was identified. Over six months, wellbeing increased in the Chinese ethnic group but not in the Western group. Interviews identified four themes: embodied breath, belonging, teacher congruence, and evaluative pressure.

**Discussion:**

The findings suggest that ethnic-cultural identity and cognitive reappraisal may help explain the observed association between vocal-training tradition and psychological wellbeing. However, the complete confounding of training tradition with institution and country prevents attributing group differences to pedagogy alone. Cross-sectional mediation models and the observational design further limit causal interpretation. These hypothesis-generating findings warrant longitudinal, multisite research to clarify the relationships among vocal training, cultural identity, emotion regulation, and wellbeing.

## 1 Introduction

While there are long-standing traditions of vocal training for its artistic and professional results, there is increasing evidence that continuous vocal training is associated with measurable benefits for mental health, both physiologically, psychologically, and socially, through mechanisms only partially understood to date. Controlled breathing and vocalization are key elements of most vocal pedagogy, which have been shown to activate the autonomic nervous system and evoke changes toward calmer, more socially-receptive states (Porges, 2007); and empirical studies are starting to document those connections directly: Breathing in the voice is entraining heart-rate variability to the phrasing of breath (Vickhoff et al., 2013); Slow controlled breathing broadly supports parasympathetic influences (Zaccaro et al., 2018); and choral singing has been correlated with decreased negative affect and altered stress-related biomarkers as compared to passive listening (Kreutz et al., 2004). Meanwhile, group singing seems to promote social bonding to a greater extent than other shared activities (Pearce et al., 2015; Weinstein et al., 2016). Thus, singing provides psychological and social wellbeing benefits that extend beyond its direct physiological effects.

However, very little attention has been paid in this literature to the potential for variation in these mechanisms across pedagogical traditions that in turn vary culturally. The majority of the literature on singing and wellbeing has focused on choral, classical or popular music contexts, but remains unclear whether music traditions with different logics, different relationships with breath and embodiment, and different social functions of singing have similar, less effective, or more beneficial mental health outcomes. Theoretically important comparison cases include Chinese ethnic singing traditions, including Mongolian long-song, Zhuang polyphonic singing and minority folk songs from the various regions of China that are taught within a conservatory context. Chinese ethnic vocal pedagogy is generally more community-oriented and cross-generational than much Western vocal pedagogy, which is based on individual technical mastery, and measured by competitive jury and audition structures, suggesting that the wellbeing associations of the teaching and learning of Chinese ethnic voice may operate through a somewhat different set of mechanisms, including those related to cultural identity and belonging (Phinney, 1990; Phinney and Ong, 2007; Smith and Silva, 2011).

Terminology. Three terms are used in this paper and they are not interchangeable. “Chinese ethnic minority vocal traditions” (shaoshu minzu shengyue) refers to the vocal repertoires, techniques and community practices associated with the officially recognized non-Han minzu of China (for example Mongolian, Zhuang, Yi, Bai and Dai), as taught in conservatory and university programs. “Chinese national singing” (minzu changfa) refers to the conservatory genre and training system that draws on Chinese folk, ethnic and operatic sources and often blends them with bel canto technique; it is not tied to one ethnic group, and students of any ethnicity, including Han students, may enroll in it. “Indigenous” is not an official or widely used classification for ethnic minorities in China and carries different historical, political and legal meanings in Australasian, North American and international frameworks of Indigeneity. We therefore do not describe our participants or settings as Indigenous: the sample consists of students and practitioners in institutional programs, not community-based knowledge holders, and “Indigenous” is used only when reporting sources that use it. In the rest of the paper, “Chinese ethnic vocal training” is shorthand for study in a program that teaches Chinese ethnic minority vocal traditions (including minzu training that blends them with bel canto), and participants’ own ethnic background is not assumed to match the tradition they study.

Another gap is in the psychological atmosphere in which training is taking place. In accordance with the self-determination theory, the satisfaction of three basic psychological needs – autonomy, competence and relatedness – is necessary for wellbeing and training environments that focus on interpersonal control and evaluation have been detrimental to the satisfaction of these needs, even if they contain similar technical content (Ryan and Deci, 2000; Bartholomew et al., 2011). Although there is well documented evidence of music performance anxiety amongst musicians in general (Kenny, 2005; Steptoe and Fidler, 1987), and amongst the professional orchestra musician there is measurable evidence of wellbeing deficits associated with the demands of the music performance culture (Kenny et al., 2014), there is a lack of comparative evidence to suggest that this evaluative burden is systematically different between western and non-western pedagogical cultures. In music education, applications of SDT have started to address the issue of motivational climate (Evans, 2015) but not yet to explore the area of cross-cultural comparison as investigated in this study.

The present study aims to address these gaps by comparing mental-health outcomes, and statistical pathways plausibly linked to them, between Western vocal training and Chinese ethnic minority vocal training. Drawing on polyvagal theory, social identity theory (SIT) and self-determination theory (SDT), we propose and test an integrative conceptual model, the Vocal Pedagogy-Psychological Wellbeing Integration Model (VP-PWIM). In the model, training tradition is hypothesized to be associated with mental wellbeing both directly and indirectly through two parallel mediators: self-reported emotion regulation and ethnic-cultural identity. Training intensity and instructor-student cultural congruence are hypothesized to moderate these pathways. Rather than comparing aggregate wellbeing scores alone, this multi-mechanism approach asks why differences may arise and when they are larger or smaller, while recognizing that the observational design cannot show that pedagogy causes them.

In addition to the psychology of music, this study has practical implications for the training of vocal pedagogists, music education mental health support in conservatories, and cross-cultural music education policy, as conservatories start to offer students non-Western vocal music classes in their curricula without a strong evidence base for the impacts of doing so on students’ wellbeing. The study also seeks to characterize both the quantitative strength of these pathways and the experience of the practitioner with regard to them, through quantitative structural modelling and qualitative thematic analysis of practitioner interviews. The remainder of this paper is organized as follows. Section 2 reviews the literature and develops the hypotheses, and Section 3 describes the methodology. Section 4 reports the quantitative and qualitative results, Section 5 discusses implications, limitations and future research, and Section 6 concludes.

## 2 Literature review

### 2.1 Western vocal training, self-efficacy, and self-esteem

Since 2020, the quantitative literature on singing and psychological wellbeing has come a long way from assertions about the impact of music to mediation models that detail the psychological processes that underlie the effects of vocal training. The most obvious one is Jiang (2024), who surveyed 326 undergraduate and postgraduate students at one of the Chinese Universities and used partial least squares structural equation modelling to test the mediating role of self-efficacy and self-esteem between music education (including vocal training) and psychological wellbeing. All hypothesized pathways were supported: Music education was positively correlated with self-esteem (*β* = 0.52) and self-efficacy (*β* = 0.50), which were in turn positively correlated with psychological wellbeing, which was in turn positively correlated with academic performance. The double mediation is important for the present study as it is found with a fitted structural modelling instead of a correlational model, and because it shows that the link between musical training and wellbeing is not direct but indirect and is mediated by internal resources (mediators) that musical training has been shaped to influence. Building on the latter, Wu and Lu (2021) traced the effects of a different sample of learners to empathy and prosocial behavior, advancing the case for self-related benefits that go beyond the instrumental and presumably influence how ensemble- and studio-based vocal pedagogies have an impact on students beyond the skill itself.

In the field of vocal training, the concept of self-efficacy has been operationalized more and more accurately. Lã et al. (2023) created and validated an instrument that measures the Self-Efficacy Beliefs in Singing Voice (Singing-VoSES), providing a voice-specific measure of self-efficacy rather than a generic measure of musical self-efficacy; this is important because self-efficacy beliefs regarding the voice are likely to be more intertwined with physical self-image and vulnerability compared with, for example, self-efficacy beliefs about piano playing. Qualitatively, Lewis and Hendricks (2022) represent the entanglement in interviews with collegiate vocalists, who consistently reported that their self-efficacy was tied to their sense of embodiment, stating that “it’s your body, it’s part of who you are,” and they identified teacher relationships, physiological states, and affective feedback loops as the prevalent influences on their confidence, highlighting Bandura’s four sources of self-efficacy but emphasizing the somatic dimension unique to voice pedagogy. This is confirmed by Bi and Rauduvaitė (2025), who interviewed university vocal educators in a Chinese higher education context, who found that manageable task design, peer observation, feedback and most importantly an emotionally safe rehearsal environment, are all important factors in the development of self-efficacy in prospective music teachers, bringing the Western literature on self-efficacy and the Chinese pedagogical context together and suggesting that some of the psychological mechanisms identified in predominantly Western samples have some generalizability to conservatory-style music teaching in China.

### 2.2 Performance anxiety as the dominant risk pathway in conservatory vocal training

The self-efficacy literature reports a protective pathway, while the performance-anxiety literature reports a risk pathway, and recent studies now begin to model both the domains within a common structure that includes an inverse relationship between the elements. A gender-based structural equation model of 526 Chinese vocal music students, used by Ye (2026), showed that social support from parents, educators and peers indirectly decreased music performance anxiety (MPA) by reducing self-efficacy, self-worth and intrinsic motivation, and that the indirect effects were stronger for female students than for male students. The model fit indices (CFI = 0.928, RMSEA = 0.061) validate the structural validity of this pathway, and the negative direct path of self-worth (*β* = −0.62 for women) and self-efficacy (*β* = −0.66 for women) towards reduced MPA indicate that the present study has a template for the type of structural pathway it proposes to test and that gender is a moderator variable which a comparative vocal-pedagogy study cannot overlook. Surveying students from the French-speaking conservatory, Racine et al. (2024) arrive at a complementary mechanism from the personality side: for “excellencism,” there is no difference in performance anxiety levels between students with and without high standards, whereas for “perfectionism,” there is a higher burden of performance anxiety among students with high standards, one that has direct implications for how feedback and evaluation are framed in vocal pedagogy, the West or the Chinese tradition.

Structure alone cannot provide that texture; it’s qualitative work. In their phenomenological study of Turkish conservatory students receiving western classical music training in vocal and instrumental music, Okan and Usta (2021) revealed that the most common causal factors of performance anxiety were jury evaluation, which led to the students’ trembling, muscle contraction and attentional narrowing, and the lack of formal training to deal with performance anxiety in the conservatories, despite the students’ awareness of the need for such training. Zhukov et al. (2026) push the measurement of MPA into the physiological domain, which combines self-report trait-anxiety measures and objective vocal-task performance (reading, sustained notes, octave leaps, and self-selected repertoire) among contemporary commercial music vocal students, and found that none of their participants met criteria for pathological anxiety, but physiological markers of arousal are detectable even in students who reported coping well — reminding us that self-report and physiological pathways can diverge, and that a study relying only on structural modelling of self-report data (as the present study does) can treat its quantitative pathways as only one of several lenses on the embodied stress response of vocal performers. Collectively, these studies support the notion that performance anxiety is the most well-documented negative mental-health pathway in vocal training research, with self-efficacy, self-worth, social support, and perfectionism as the major factors that predict it; however, all samples come from Western classical or Chinese conservatory settings employing what has been described as broadly Western pedagogies, a limitation that means we cannot conclude whether the anxiety architecture present in ethnic practices with their distinct aesthetic criteria and evaluative norms is similar.

### 2.3 Chinese ethnic vocal pedagogy: a technically rich but psychologically thin literature

Literature on Chinese ethnic and national vocal pedagogy has gained a certain level of development regarding the technique, while for mental health it is relatively silent – and this imbalance is worthwhile to be noted in the present review. Comparative acoustic and pedagogical scholarship has always been used to regard Chinese national singing as an intermediate form of singing between Western bel canto and popular singing. In a comparative study of the traditional Chinese style and Western bel canto, Pan (2023) records that both styles require strict diction and proper breath management, but that the standards for diction are opposite; whereas with bel canto, the idea of diction is to achieve a clear, focused, bright tone that resonates in the vernacular, the traditional Chinese style aims for the opposite, a chiaroscuro resonance balance, which is more typically Italianate. This is confirmed acoustically: Liu and Wallmark (2024) found timbral features (spectral centroid, roughness, inharmonicity) to be useful in distinguishing the different roles to be sung in Peking opera, and therefore that the acoustic production of Peking opera singing is internally consistent and does not rely on a Western template. This body of work is important for the mental-health question in this study because it has been shown that the two traditions are not only different in terms of ornamentation, but also in the physiological and acoustic goals singers are trained to achieve, and thus, comparisons of their mental-health effects must take this difference into account, as the technical demands, vocal loads, and evaluative criteria have not been the same.

This literature does not yet provide empirical evidence, however, that these pedagogical and acoustic differences lead to psychological outcomes, such as anxiety, self-efficacy, or wellbeing. The studies available on the integration of bel canto technique into Chinese vocal training only addresses the pedagogical and cultural negotiation process involved in integrating the two systems (breath mechanics, resonance placement, diction training), while not quantifying the mental-health outcomes of students based on those two systems, respectively, or the combination of the two systems. This is actually a meaningful gap: In the Western-classical or generally “Chinese higher music education” samples used in the studies mentioned in sections 1 and 2 above, the difference in the self-efficacy or anxiety levels caused by the ethnic/national vocal training and bel canto-based training has been measured almost exclusively without breaking down the former into its various components, leaving it as an open empirical question, as this study is designed to address, whether the different technical demands, aesthetic values, and cultural ascriptions of ethnic vocal training lead to a distinct self-efficacy or anxiety pattern as compared to the bel canto based training, or whether the psychological mechanisms described above apply equally to both traditions.

### 2.4 Cultural identity, ethnic music and psychological wellbeing

Other work, however, is concerned with ethnic and folk music and its connection with psychological well-being, from a cultural identity perspective, and in this domain, although not so informative as the work on vocal pedagogy outlined above, Chinese-context studies are more informative than other traditions. Huang et al. (2025) examined 1,516 Chinese youths and found that through serial mediation, cultural participation, such as musical participation, enhances psychological well-being via appreciation of beauty, gratitude, hope, and a Buddhist construct that was secularized, namely ‘nonattachment’. One of the few studies that modelled a full mediation chain from cultural engagement to wellbeing, in a specifically Chinese cultural-psychological framework, and the results showed that active participation had both mediated and direct wellbeing effects, directly relevant to a study comparing western and ethnic Chinese vocal training, due to the results indicating that culturally embedded musical practice may operate through pathways of wellbeing (meaning, transcendence, cultural belonging) which generic self-efficacy models do not capture. The findings by Chen et al. (2025) are converging in another population and method: the pre–post educational intervention with older adults in Chongqing, in classical Guqin listening and reflective dialogue, was responsible for significant reductions in depression (*t* = 3.21, *p* < 0.01) and anxiety (*t* = 2.94, *p* < 0.05) and qualitative data suggested that the mechanisms at work included cultural identity, self-awareness and resilience. This study is of instrumental folk music and the elderly population; however, it provides a rare example of a mixed-methods design (pre–post measures of symptoms, with qualitative interview data) similar to the mixed-methods design proposed in the present study, and suggests that culturally specific music engagement can be accompanied by measurable change in depression and anxiety, not only by self-reported benefit.

The mental-health value of culturally embedded musical practice, such as practice based on indigenous or traditional vocal and musical forms, cannot be simplified to the acoustic or technical characteristics of the music itself, but is linked to a broader body of knowledge that is necessarily embedded in culture. Building on initial findings from the CHIME project in The Gambia (reduced antenatal anxiety and depression after musical group engagement), the authors of Sigwebela et al. (2026) undertook formative qualitative studies in South Africa to discover how community-based musical practices associated with specific ethnic groups (Nguni, Sotho, Tswana, Tsonga, and Venda) were used to process hardship, foster social cohesion and promote perinatal mental health. This cross-cultural evidence points to a common theme: that ethnic and traditional musical participation has a strong link to psychological wellbeing, primarily through cultural-identity and belonging mechanisms which are conceptually different from, but not mutually exclusive to, the self-efficacy and performance-anxiety mechanisms that are prevalent in the Western conservatory literature surveyed in Sections 1 and 2. However, none of the studies reviewed test both mechanism sets – technical self-efficacy/anxiety pathways and cultural-identity/belonging pathways – in the same sample of trained voices, the specific comparison that is the basis of the structural model used in the present study.

### 2.5 Synthesis: convergence, divergence, and the gap this study addresses

When these 14 studies are read together, these three conclusions follow that directly justify the design of the present study. First, there are reasonably well-specified pathways, based upon self-report evidence, that connect musical and vocal training to mental health, and these data are increasingly complemented by physiological and qualitative data (Okan and Usta, 2021; Racine et al., 2024; Zhukov et al., 2026; Ye, 2026): self-efficacy, self-worth, social support, and perfectionism orientation converge to performance anxiety and psychological wellbeing broadly. Second, the literature of Chinese ethnic and national vocal pedagogy is technically and acoustically advanced (Pan, 2023; Liu and Wallmark, 2024), but has not yet been directly linked with these same psychological factors, which leaves the field without any empirical comparative data to determine if ethnic vocal training yields a distinct self-efficacy and anxiety outcome as compared to bel canto vocal training. Third, the self-efficacy/anxiety framework which is prevalent in the literature of conservatories does not squarely engage with the wellbeing literature on culturally embedded musical participation, which is a parallel body of literature that shows that such musical participation has wellbeing benefits that run and are distinct from the self-efficacy/anxiety framework (Huang et al., 2025; Chen et al., 2025; Sigwebela et al., 2026).

Thematically, the literature also comes together around the benefits of integrating structural equation modelling with qualitative or interview data, with few individual studies doing this. Each of the above authors—Jiang (2024), Ye (2026), and Huang et al. (2025)—shows the explanatory power of SEM in isolating mediating psychological pathways in a music-and-wellbeing context; and Lewis and Hendricks (2022), Okan and Usta (2021), Bi and Rauduvaitė (2025), and Chen et al. (2025) show that the embodiment, teacher-relationship, and cultural-meaning dimensions emerge in the results of qualitative and interview-based methods, dimensions that are unable to be designed into structural models starting from pre-specified latent constructs. The present study’s proposed combination is just such: a structural model that has been tested to see if the self-efficacy/anxiety pathway that has been found in Western and Chinese conservatory research holds for ethnic vocal training, and thematic analysis of practitioner interviews that surfaces the cultural-identity and belonging mechanisms that the quantitative literature on ethnic music (Huang et al., 2025; Chen et al., 2025) suggests are active but that no existing vocal-pedagogy study has measured directly. According to the study’s rationale, while conservatories are embracing non-Western vocal traditions into curricula, there is no evidence base to support the research on student wellbeing, and since the study’s purpose is to cast light on the gap between the two and the mechanisms through which a mixed-methods comparative design is best suited to adjudicate, it is no surprise that the literature reviewed is comparative and synthesized.

### 2.6 Hypothesis development

The hypotheses of this study are based on three related theories that, together, describe how the vocal training tradition differentially impacts mental wellbeing: polyvagal theory, social identity theory, and self-determination theory. Theories are linked to different causal mechanism in the proposed VP-PWIM framework, and both theories provide support for the mediation and moderation pathways investigated in this study.

The first theoretical foundation is polyvagal theory, which states that controlled breathing, vocalizing and vocal prosody activate the myelinated vagus nerve and the social engagement system and cause a shift toward a calm, socially engaged autonomic nervous system (Porges, 2001, 2003, 2007). There is empirical evidence to suggest that structured vocal practice is linked with autonomic regulation: singing has been found to influence autonomic regulation in terms of heart-rate variability related to breath phrasing (Vickhoff et al., 2013), and controlled, slow breathing, in general, has been shown to influence autonomic regulation in terms of activation of the parasympathetic nervous system and improvements in emotion regulation (Zaccaro et al., 2018). In choir singing, specifically, negative affect has been found to decrease more than passive listening, and cortisol and immune markers have been found to change in a manner that is plausibly related to the synchronized breathing and singing (Kreutz et al., 2004), whereas group singing has been shown to speed up social bonding, plausibly through the mechanisms associated with synchronized breathing and singing (Pearce et al., 2015; Weinstein et al., 2016). There is also evidence of individual variations in emotion-regulation style and the reappraisal processes potentially engaged by sustained vocal training, also linked to wellbeing outcomes (Gross and John, 2003). Since Chinese ethnic vocal pedagogy generally incorporates breath-based technique into group-based, ritualized practice that is introduced at an early age, it is hypothesized that this is associated with stronger self-reported emotion regulation than in Western training. Polyvagal theory motivates this hypothesis, but the present study measures regulation only with the reappraisal subscale of the Emotion Regulation Questionnaire, which assesses self-reported cognitive strategies; it does not measure vagal, parasympathetic or respiratory activity, so the polyvagal mechanism itself is not tested. This rationale leads to the following hypotheses: Hypothesis 2a: Training tradition is a significant predictor of emotion regulation; Hypothesis 3a: Emotion regulation is a significant predictor of mental wellbeing; Hypothesis 4a: The relationship between training tradition and mental wellbeing is indirect through emotion regulation; and Hypothesis 7: Training intensity moderates the tradition-wellbeing pathway through emotion regulation.

The second theoretical underpinning is social identity theory (SIT) (Tajfel and Turner, 1979), which was extended by Phinney’s model on ethnic identity development, which suggests that the development of a secure and achieved sense of belonging to a valued in-group is a powerful source of self-esteem and psychological wellbeing (Phinney, 1990; Phinney and Ong, 2007). In a meta-analysis of 184 studies, ethnic identity had a strong, but not overly strong, correlation to personal wellbeing, particularly with positive indicators of wellbeing among people of color, as compared to distress indicators (Smith and Silva, 2011). In the case of Chinese ethnic repertoire, which explicitly references an anchor to certain ethnic groups, regions and communities, singing is explicitly theorized as an identity-affirming practice, which is of the nature that this literature associates with wellbeing, which may foster ethnic-cultural identity to a degree not reached by generic technical training. These arguments yield Hypothesis 2b: Training tradition predicts ethnic-cultural identity, Hypothesis 3b: Ethnic-cultural identity predicts mental wellbeing, Hypothesis 4b: Ethnic-cultural identity mediates the tradition-wellbeing relationship, and Hypothesis 5: Instructor-student cultural congruence moderates the indirect association between training tradition and mental wellbeing through ethnic-cultural identity.

The third theoretical underpinning is self-determination theory, which assumes that psychological wellbeing is dependent upon meeting the three basic needs of autonomy, competence, and relatedness (Ryan and Deci, 2000). These needs are thwarted in training environments by interpersonal control, which is linked to reduced functioning such as burnout and negative affect, albeit when the technical content is similar (Bartholomew et al., 2011). This dynamic has been well documented in music performance specifically: music performance anxiety is common amongst musicians and there are systematic relationships between evaluative context and anxiety (Kenny, 2005; Steptoe and Fidler, 1987), professional orchestral musicians exhibit measurable psychological wellbeing deficits as a result of the demands of their training and performance culture (Kenny et al., 2014), and self-determination theory has been directly applied to music education to explain how autonomy-supportive versus controlling instructional climates relate to motivation and persistence in music education (Evans, 2015). The composite wellbeing outcome of this study is based on the model of psychological wellbeing developed by Ryff (1989), which comprises six dimensions: autonomy, environmental mastery, personal growth, positive relations with others, purpose in life, and self-acceptance. The PHQ-9 depression measure was used alongside this measure and is a brief validated depression severity indicator with established psychometric properties (Kroenke et al., 2001). The logic behind this leads to Hypothesis 1: Training tradition is associated with overall mental wellbeing, and Hypothesis 6: The direct relationship between the training tradition and the mental wellbeing is moderated by the training intensity.

The three theories, taken together, suggest a multi-mechanism model in which the three mechanisms (physiological regulation, sociocultural identity, basic psychological need satisfaction) act in parallel, but theoretically independent pathways to mental wellbeing as a result of vocal training tradition, yielding convergent theoretical support for the hypotheses tested in this study (Hypotheses 1–7) (Figure 1).

**Figure 1:**
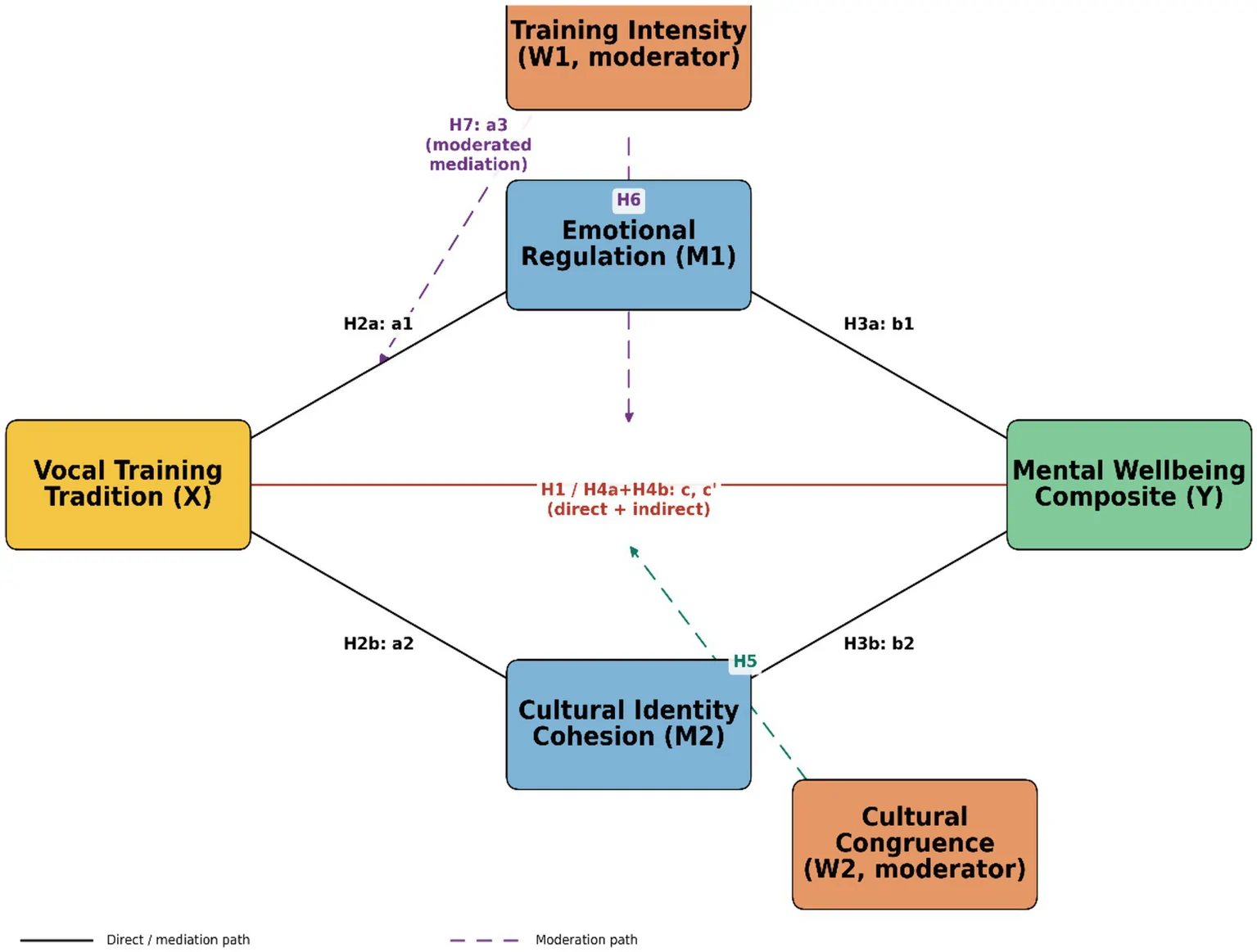
Conceptual framework diagram.

## 3 Research methodology

This study uses a convergent parallel mixed-methods design in which a quantitative strand and a qualitative strand were collected and analyzed in parallel and then integrated through a joint display. The quantitative strand is an observational, between-groups comparative survey of two vocal pedagogical traditions: Western classical/contemporary vocal training and Chinese ethnic minority vocal training (minzu shengyue). Participants were not assigned to traditions and no intervention was delivered, so the design is not quasi-experimental; because tradition is confounded with institution, region and country, group differences are interpreted as associations rather than as effects of pedagogy. A six-month longitudinal sub-study describes change in a random subsample and is analyzed separately (Table 1); all mediation and moderation models use baseline (T1) data only and are therefore cross-sectional. In the qualitative strand, semi-structured interviews contextualize the numerical findings in terms of lived pedagogical and cultural experience. The design rests on the view that constructs such as anxiety, self-esteem and ethnic-cultural identity may not be fully captured by psychometric measures alone when two training systems operate within different pedagogical and communal frameworks.

**Table 1:** Longitudinal change in wellbeing and depressive symptoms (T1–T3).

Outcome	Group/effect	T1 (0 m) M (SD) or 95% CI	T2 (3 m) M (SD) or *z*	T3 (6 m) M (SD) or *p*
Wellbeing composite (z)	Western	−0.20 (0.66)	−0.17 (0.68)	−0.17 (0.72)
	Chinese ethnic	0.22 (0.81)	0.35 (0.82)	0.50 (0.82)
	Time (per month), Western: *B* = 0.004, SE = 0.003	[−0.002, 0.010]	*z* = 1.33	*p* = 0.184
	Tradition at T1: *B* = 0.412, SE = 0.109	[0.199, 0.624]	*z* = 3.79	*p* < 0.001
	Time × Tradition: *B* = 0.042, SE = 0.004	[0.034, 0.050]	*z* = 10.06	*p* < 0.001
PHQ-9	Western	6.4 (3.4)	6.3 (3.9)	6.7 (4.2)
	Chinese ethnic	5.3 (3.4)	4.6 (3.4)	3.9 (3.4)
	Time (per month), Western: *B* = 0.047, SE = 0.031	[−0.013, 0.107]	*z* = 1.53	*p* = 0.127
	Tradition at T1: *B* = −1.047, SE = 0.520	[−2.065, −0.028]	*z* = −2.01	*p* = 0.044
	Time × Tradition: *B* = −0.276, SE = 0.043	[−0.361, −0.191]	*z* = −6.36	*p* < 0.001

Complete cases, *n* = 192 (96 per tradition). Linear mixed models with random participant intercepts; time in months.

The stratified purposive sampling method was used to sample the participants from the two pedagogical traditions within their institutions. *A priori* sample size was determined using G*Power 3.1.9.7 for a two-group MANOVA with mediation and moderation extensions with a medium effect size (*f* = 0.25), α = 0.05, power (1-β) = 0.80 and four outcome variables, resulting in a minimum required sample of *N* = 396. The number of subjects (N) was planned to be 480, with two equal-sized groups (*n* = 240 each) of training traditions and a 1:1:1 ratio of training level (novice = 1–2 years, intermediate = 3–5 years, advanced = 6 + years), to provide a margin of approximately 18% for attrition in the longitudinal sub-study and for subgroup regression stability.

The Western vocal training group (*n* = 240) consisted of students from three conservatory-affiliated vocal training programs: the New England Conservatory of Music (Boston, USA, *n* = 80); the Royal College of Music (London, United Kingdom, *n* = 80); and the University of Music and Performing Arts Vienna (Vienna, Austria, *n* = 80). The Chinese ethnic vocal pedagogy group consisted of 240 participants from four institutions that specialize in minority vocal traditions, including Ethnic Vocal Music Department of the Central Conservatory of Music (Beijing, *n* = 60, Han and multi-ethnic bel canto-minzu training, *n* = 60), Yunnan Arts University (Kunming, Yunnan Province, *n* = 60, focused on ethnic vocal traditions of Yi, Bai and Dai), Guangxi Arts University (Nanning, Guangxi Zhuang Autonomous Region, *n* = 60, specializing in the traditional ethnic vocal of the Zhuang people including the shan’ge (mountain song) tradition), and Inner Mongolia Normal University (Hohhot, Inner Mongolia, *n* = 60, focused on Mongolian long song urtiin duu and khoomei singing). Inclusion criteria for subjects were: 18–45 years of age; participation for at least 1 year in or professional involvement with their own vocal tradition; no pre-existing psychiatric disorder, diagnosed and needing active pharmacological treatment at the time of recruitment. Recruitment was done by institutional gatekeepers (department chairs and studio instructors) via proportional quota sampling to maximize gender balance (50/50) and balance in training level as well as in the Chinese subsample, in ethnic-tradition subgroups. The Chinese group comprises students enrolled in programs that teach minority vocal traditions; the Beijing program blends bel canto and minzu training and enrolls Han and multi-ethnic students.

The present study is based on an original integrative model of the relationship between pedagogical tradition and mental-health outcomes, the Vocal Pedagogy-Psychological Wellbeing Integration Model (VP-PWIM). The model specifies parallel statistical mediating pathways and moderation by cultural congruence and training intensity; these paths are tested as statistical associations, not as causal mechanisms. Vocal training tradition is the independent variable (X), a categorical variable dummy-coded 0 = Western, 1 = Chinese ethnic. The outcomes (Y) were measured with the following validated instruments, each administered with the items listed: the 42-item short form of Ryff’s Scales of Psychological Well-Being (six 7-item dimensions; total score 42–252), the 9-item Patient Health Questionnaire (PHQ-9; depressive symptoms), the 20-item trait subscale of the State–Trait Anxiety Inventory, Form Y (STAI-T; trait anxiety) and the 10-item Rosenberg Self-Esteem Scale (RSES). The mental-wellbeing composite is the unweighted mean of four scale scores, each z-scored on the full sample of 480 using the sample standard deviation: z(Ryff total), z(RSES total), −z(PHQ-9) and −z(STAI-T), so that higher values indicate better mental wellbeing and each scale contributes equally; it is not a factor score. A scoring audit recomputed every scale score from the item-level data and confirmed that no item was dropped from any scale (maximum difference from the stored scores 0.0003, attributable to rounding). Two parallel mediators were specified. M1, emotion regulation, was measured with the six-item cognitive reappraisal subscale of the 10-item Emotion Regulation Questionnaire (ERQ-R; mean score, 1–7); the four expressive suppression items were not used. It captures self-reported use of reappraisal and does not measure vagal, parasympathetic, respiratory or other physiological regulation; polyvagal theory therefore motivates the hypotheses but is not tested, and the M1 pathway is interpreted only as an association involving self-reported emotion regulation. M2, ethnic-cultural identity, was measured with the six-item Multigroup Ethnic Identity Measure-Revised (MEIM-R; 1–5), which assesses exploration and commitment in relation to one’s own ethnic group. We label it ethnic-cultural identity rather than cohesion because the MEIM-R does not measure cohesion as such. Its meaning may differ between groups, because respondents rate their own ethnic or cultural group and that referent may differ between a Chinese group studying minority repertoire and an internationally diverse Western group; measurement invariance across groups was not tested, so group comparisons on M2 are interpreted cautiously. Two moderators were included: W1 = training intensity (weekly hours of practice, continuous) and W2 = instructor-student cultural congruence (a 1–7 Likert composite of perceived congruence between the practitioner’s cultural background and the cultural origin of the tradition).

The primary analytic strategy employs Hayes’ PROCESS macro (Model 4 for parallel mediation, Model 59 for moderated mediation) implemented in R (lavaan and mediation packages) alongside multilevel modelling to examine the nested structure of participants within institutions. Because PROCESS-type models do not model clustering, the mediation and moderated-mediation estimates treat participants as independent (ordinary least squares with HC3 robust standard errors), and the multilevel models below and the cluster-robust analyses described next were used to check how clustering by institution affects the estimates. Because only seven institutions were sampled, cluster-robust inference was also applied (see Robustness and sensitivity analyses below). The baseline regression equation is specified as:

$$Y=\beta_{0}+\beta ₁X+\beta ₂W1+\beta ₃W2+\beta ₄(X\times W1)+\beta_{5}(X\times W2)+\varepsilon$$

where Y is the composite wellbeing outcome, X is training tradition, and the interaction terms (X × W1, X × W2) test moderation effects. The parallel mediation pathways are estimated through the following equation system:

$$M1=a_{₁}X+e_{₁}M2=a_{₂}X+e_{₂}Y=c^{\prime }X+b_{₁}M1+b_{₂}M2+e₃$$

with the indirect effects computed as a₁b₁ (emotion regulation pathway) and a₂b₂ (cultural identity pathway), and the total effect decomposed as c = c′ + a₁b₁ + a₂b₂. Bias-corrected bootstrapped confidence intervals (10,000 resamples) are used to test the significance of indirect effects, given the non-normality typically observed in psychometric mediation data.

For the moderated mediation component, conditional indirect effects are modelled as:

$$\begin{array}{c} M1=a_{₁}X+a_{2}W1+a_{₃}(X\times W1)+e_{₁}Y=c^{\prime }X+b_{₁}M1+b_{₂}W1\\ +b_{₃}(X\times W1)+e_{₂} \end{array}$$

Index of moderated mediation (IMM) = a₃b₁, tested for significance via bootstrapped confidence intervals; a non-zero IMM indicates that the strength of the emotion-regulation mediating pathway varies systematically as a linear function of training intensity; curvilinear moderation was examined separately in the robustness analyses.

To account for the hierarchical nesting of individual participants (Level 1) within institutions (Level 2), a multilevel (hierarchical linear) model is specified as:

$$\begin{array}{l} \text{Level}\;1:Y_{\mathrm{ij}}=\beta_{0\;\;j}+\beta_{1}X_{i\;\;j}+r_{i\;\;j}\;\text{Level}\;2:\beta_{0\;\;j}=\gamma_{0\;\;0}\\ +\gamma_{0\;\;1}{\left(\text{Institution Type}\right)}_{j}+u_{0\;\;j}\;\beta_{1\;\;j}=\gamma_{1\;\;0}+u_{1\;\;j} \end{array}$$

where γ₀₁ captures institution-level variance attributable to broader cultural-pedagogical context beyond individual training tradition, and u₀ⱼ, u₁ⱼ represent random intercept and slope variance components, with intraclass correlation coefficients (ICC) computed to justify multilevel treatment over ordinary least squares.

Robustness and sensitivity analyses. (a) Cluster-robust inference: path coefficients were re-estimated with CR1 cluster-robust standard errors for the seven institutions, evaluated against a t distribution with 6 degrees of freedom, and with Webb wild-cluster bootstrap *p*-values, which behave better than conventional cluster-robust tests when clusters are few; indirect effects and indices of moderated mediation were also bootstrapped by resampling whole institutions. With three and four institutions per tradition, institution-level resampling can give unstable and artificially narrow intervals, so the wild-cluster *p*-values are taken as the primary clustered inference and the cluster-bootstrap intervals as sensitivity checks. (b) Latent growth models (three waves; tradition predicting intercept and slope, with time coded per 3 months) were fitted separately for the wellbeing composite and the PHQ-9 and checked against random-slope mixed models. (c) Cross-lagged panel models (T1 to T2 and T2 to T3) examined whether wellbeing and PHQ-9 predicted each other over time. (d) Curvilinear moderation was tested by adding X × W^2^ terms for each moderator to the outcome and mediator models, testing the added variance against the linear model, and, for each group, checking whether the slope was significantly positive at the observed minimum and significantly negative at the observed maximum of the moderator (an inverted-U pattern).

All psychological assessments were independently translated and back-translated from English to Mandarin Chinese according to World Health Organization recommendations, with disagreements settled through bilingual review panels to achieve semantic and conceptual equivalency between English and Chinese versions. Data collection took place in three stages over a period of 6 months, namely baseline (T1), three-month follow-up (T2), and six-month follow-up (T3). A random 40% subsample (*n* = 192; 96 per group) was designated for follow-up. Semi-structured interviews were conducted on a purposive sub-sample at T3 (*n* = 40: five participants at each of the four Chinese institutions and six or seven at each of the three Western institutions, giving 20 per tradition), examining subjective experiences of breathing exercises, vocal tonality, communal performance, and cultural identification. Interviews were transcribed verbatim and analyzed with codebook (coding-reliability) thematic analysis, organized around Braun and Clarke’s six phases. Because two coders applied a shared codebook independently and agreement was quantified with Cohen’s kappa, we do not describe the analysis as reflexive thematic analysis, which treats coding as an interpretive practice and does not use inter-coder reliability statistics. Themes were then compared with the quantitative results through a joint display (Table 2). Ethics and data governance. Ethical clearance was obtained from the ethics committees of the participating universities for each site. All participants gave written informed consent, in the language of their choice. Confidentiality was maintained in line with the GDPR and China’s Personal Information Protection Law (PIPL), using pseudonymized coded datasets stored on encrypted institutional servers.

**Table 2:** Joint display matrix: integration of qualitative themes with quantitative results.

Theme	Qualitative finding	Quantitative result	Integration
1. Breath as embodied calm	Breath control described as a way to settle the body, especially in sustained group phrasing	Tradition → ERQ-R a₁ = 0.418; ERQ-R → wellbeing b₁ = 0.234; indirect a₁b₁ = 0.098 [0.060, 0.147] (Table 8); index of moderated mediation for practice hours = 0.006 [0.001, 0.012] (Table 10)	Convergence: interviews offer a possible account of how self-reported regulation develops, and that more practice strengthens it
2. Belonging and ancestral continuity	Songs affirm family, language and place; strongest in Chinese ethnic participants	MEIM-R d = 0.78 (Table 4); a₂ = 0.425; b₂ = 0.326; indirect a₂b₂ = 0.139 [0.089, 0.201] (Table 8)	Convergence: the largest group difference and the larger indirect pathway match the most group-specific theme
3. Cultural congruence and teacher relationship	Congruence valued, but inseparable from warmth and autonomy; outsider teachers questioned	X × W2 β = 0.122, *p* = 0.061 (Table 7); index for congruence = 0.025 [−0.004, 0.063] (Table 10)	Divergence: participants stress congruence, yet the scale did not show significant moderation; one possible reason, to be tested, is that congruence is blended with teacher climate
4. Evaluative pressure and burden of intensity	Juries and ranking produce anxiety, more so in Western participants; intensity can exhaust	STAI-T *d* = −0.29; PHQ-9 *d* = −0.27 (Table 4); X × W1 *β* = 0.019, *p* = 0.108 (Table 7); longitudinal time × tradition interaction (Table 1)	Partial convergence: anxiety gap is consistent, while the mixed Beijing accounts add nuance to the intensity result

## 4 Results

Participant characteristics are presented in Table 3. The 480 participants (240 per tradition) did not differ in gender, age, training level, years of training, weekly practice hours, or perceived cultural congruence (all *p*s > 0.08), indicating that the quota sampling produced comparable groups. All scales showed good internal consistency (α = 0.83–0.94; Table 4). Chinese ethnic vocal trainees reported higher psychological wellbeing (*M* = 157.7 vs. 145.2, *d* = 0.58) and self-esteem (*d* = 0.49), and lower depressive symptoms (*d* = −0.27) and trait anxiety (*d* = −0.29). They also scored higher on emotion regulation (*d* = 0.52) and, most markedly, ethnic-cultural identity (*d* = 0.78), with all differences surviving Holm correction. As Table 5 shows, the wellbeing composite correlated positively with tradition (*r* = 0.26), emotion regulation (*r* = 0.32), and ethnic-cultural identity (*r* = 0.32).

**Table 3:** Participant characteristics by vocal training tradition.

Characteristic	Western (*n* = 240)	Chinese ethnic (*n* = 240)	Test statistic	*p*
Female, *n* (%)	120 (50.0)	120 (50.0)	χ^2^ = 0.00	1.000
Age (years), M (SD)	28.7 (6.1)	29.0 (6.3)	t = 0.50	0.617
Novice (1–2 years), *n* (%)	81 (33.8)	80 (33.3)	χ^2^ = 0.04	0.981
Intermediate (3–5 years), *n* (%)	81 (33.8)	80 (33.3)		
Advanced (6 + years), *n* (%)	78 (32.5)	80 (33.3)		
Training years, *M* (SD)	4.8 (3.4)	5.0 (3.4)	*t* = 0.41	0.680
W1 Training intensity (h/week), *M* (SD)	9.9 (5.7)	9.7 (5.4)	*t* = −0.34	0.730
W2 Cultural congruence (1–7), *M* (SD)	5.0 (1.0)	5.2 (1.1)	*t* = 1.72	0.085

Continuous variables compared with Welch *t*-tests; categorical variables with Pearson χ^2^.

**Table 4:** Reliability, descriptive statistics and group comparisons on study measures.

Measure	*α*	Western *M* (SD)	Chinese ethnic *M* (SD)	Welch *t*	*p*	*p* (Holm)	Cohen’s *d* [95% CI]
Psychological wellbeing (Ryff, 42–252)	0.94	145.2 (19.6)	157.7 (23.3)	6.37	<0.001	<0.001	0.58 [0.40, 0.76]
Depressive symptoms (PHQ-9, 0–27)	0.83	6.20 (3.30)	5.31 (3.37)	−2.93	0.004	0.004	−0.27 [−0.45, −0.09]
Trait anxiety (STAI-T, 20–80)	0.91	46.1 (8.1)	43.7 (8.6)	−3.19	0.001	0.003	−0.29 [−0.47, −0.11]
Self-esteem (RSES, 10–40)	0.86	29.1 (4.1)	31.1 (4.2)	5.37	<0.001	<0.001	0.49 [0.31, 0.67]
Wellbeing composite (*z*-mean)	—	−0.20 (0.67)	0.20 (0.77)	6.00	<0.001	<0.001	0.55 [0.37, 0.73]
M1 Emotion regulation (ERQ-R, 1–7)	0.86	4.69 (0.80)	5.11 (0.80)	5.71	<0.001	<0.001	0.52 [0.34, 0.70]
M2 Ethnic-cultural identity (MEIM-R, 1–5)	0.84	3.30 (0.57)	3.73 (0.53)	8.50	<0.001	<0.001	0.78 [0.59, 0.96]

Positive *d* = higher in the Chinese ethnic group. Holm adjustment across the seven comparisons.

**Table 5:** Pearson correlations among the main study variables.

Variable	1	2	3	4	5	6	7	8
1. Tradition (X)	—							
2. ERQ-R (M1)	0.25***	—						
3. MEIM-R (M2)	0.36***	0.12**	—					
4. Intensity (W1)	−0.02	0.17***	0.05	—				
5. Congruence (W2)	0.08	0.01	0.16***	−0.04	—			
6. Wellbeing composite	0.26***	0.32***	0.32***	0.16***	0.08	—		
7. PHQ-9	−0.13**	−0.18***	−0.18***	−0.13**	−0.05	−0.70***	—	
8. STAI-T	−0.14**	−0.18***	−0.25***	−0.03	−0.03	−0.70***	0.28***	—

*N* = 480. Tradition: 0 = Western, 1 = Chinese ethnic. *p < 0.05, **p < 0.01, ***p < 0.001.

After adjustment for gender, age, and training level, the omnibus MANOVA indicated a significant tradition effect on the four mental-health outcomes [Wilks’ Λ = 0.909, *F*(4, 471) = 11.82, *p* < 0.001]. Table 6 shows that the adjusted advantage of the Chinese ethnic group was 12.52 points on psychological wellbeing (partial η^2^ = 0.082) and 2.04 points on self-esteem. It was −0.89 points on depressive symptoms and −2.46 points on trait anxiety. In the regression models (Table 7), tradition was associated with the wellbeing composite (*β*₁ = 0.397, *p* < 0.001; *R*^2^ = 0.070), supporting Hypothesis 1. Adding the moderators raised *R*^2^ to 0.113, yet neither training intensity (*β* = 0.013, *p* = 0.120) nor cultural congruence (*β* = −0.016, *p* = 0.723) had a significant main effect. The interactions with tradition were also non-significant for training intensity (*β* = 0.019, *p* = 0.108) and cultural congruence (*β* = 0.122, *p* = 0.061), so Hypothesis 6 was not supported.

**Table 6:** Covariate-adjusted group differences on mental-health outcomes (ANCOVA).

Outcome	Adj. difference (Chinese − Western)	95% CI	*F*	*p*	*p* (Holm)	Partial η^2^
Ryff psychological wellbeing	12.52	[8.75, 16.29]	*F*(1, 474) = 42.53	<0.001	<0.001	0.082
PHQ-9 depressive symptoms	−0.89	[−1.48, −0.29]	F(1, 474) = 8.62	0.003	0.003	0.018
STAI-T trait anxiety	−2.46	[−3.96, −0.96]	F(1, 474) = 10.43	0.001	0.003	0.022
Rosenberg self-esteem	2.04	[1.30, 2.78]	F(1, 474) = 29.54	<0.001	<0.001	0.059
Wellbeing composite	0.40	[0.27, 0.53]	F(1, 474) = 37.59	<0.001	<0.001	0.073

Adjusted for gender, age and training level. Omnibus MANOVA (Ryff, PHQ-9, STAI-T, RSES), tradition effect: Wilks’ Λ = 0.909, *F*(4, 471) = 11.82, *p* < 0.001.

**Table 7:** Regression of wellbeing on tradition, training intensity, cultural congruence and interactions.

Predictor	Model 1: B (SE)	Model 2: B (SE)	Model 2 *t*	Model 2 *p*	Model 2 95% CI
Intercept (β₀)	−0.199*** (0.044)	−0.201*** (0.043)	−4.63	<0.001	[−0.286, −0.116]
Tradition, X (β₁)	0.397*** (0.066)	0.394*** (0.066)	5.98	<0.001	[0.265, 0.523]
Training intensity, W1 (β₂)	—	0.013 (0.009)	1.55	0.120	[−0.004, 0.030]
Cultural congruence, W2 (β₃)	—	−0.016 (0.045)	−0.35	0.723	[−0.105, 0.073]
X × W1 (β₄)	—	0.019 (0.012)	1.61	0.108	[−0.004, 0.043]
X × W2 (β₅)	—	0.122 (0.065)	1.87	0.061	[−0.006, 0.250]
*R*^2^ / adjusted *R*^2^	0.070/0.068	0.113/0.104			
*F* (df)	35.90 (1, 478)	14.29 (5, 474)			

Outcome = wellbeing composite. W1 and W2 grand-mean centered. HC3 robust standard errors. ***p < 0.001.

The parallel mediation model (Table 8) supported both mediating pathways. Chinese ethnic training was associated with higher emotion regulation (a₁ = 0.418) and ethnic-cultural identity (a₂ = 0.425), and both mediators were associated with wellbeing (b₁ = 0.234; b₂ = 0.326; all ps < 0.001), consistent with Hypotheses 2a, 2b, 3a, and 3b. The total statistical indirect effect was 0.236 (95% BC CI [0.175, 0.313]), composed of an emotion-regulation pathway (a₁b₁ = 0.098, [0.060, 0.147]) and an ethnic-cultural identity pathway (a₂b₂ = 0.139, [0.089, 0.201]), supporting Hypotheses 4a and 4b. The direct effect was significant under conventional standard errors (c′ = 0.161, *p* = 0.018) but not under cluster-robust inference (Table 9), so we do not claim partial mediation. Because tradition, mediators and outcome were measured concurrently at T1, these estimates describe patterns of association that are consistent with, but not evidence of, temporal or causal mediation, and they treat participants as independent of institution. The identity pathway carried about 59% of the total indirect effect and the regulation pathway 41%, although the contrast between them was not significant (−0.041, [−0.115, 0.032]).

**Table 8:** Parallel mediation of the tradition–wellbeing relationship (Model 4).

Path / effect	Estimate (B)	Boot SE	95% BC bootstrap CI	*p* (paths)/CI excludes 0 (indirect)
a₁: X → M1 (ERQ-R)	0.418	0.073	[0.274, 0.559]	<0.001
a₂: X → M2 (MEIM-R)	0.425	0.050	[0.329, 0.521]	<0.001
b₁: M1 → Y (controlling X, M2)	0.234	0.038	[0.160, 0.308]	<0.001
b₂: M2 → Y (controlling X, M1)	0.326	0.059	[0.210, 0.444]	<0.001
c′: X → Y (direct)	0.161	0.069	[0.026, 0.294]	0.018
c: X → Y (total)	0.397	0.066	[0.266, 0.528]	<0.001
Indirect a₁b₁ (via emotion regulation)	0.098	0.022	[0.060, 0.147]	Yes
Indirect a₂b₂ (via ethnic-cultural identity)	0.139	0.028	[0.089, 0.201]	Yes
Total indirect (a₁b₁ + a₂b₂)	0.236	0.035	[0.175, 0.313]	Yes
Contrast (a₁b₁ − a₂b₂)	−0.041	0.038	[−0.115, 0.032]	No

Unstandardized coefficients; Y = wellbeing composite (z-units). 10,000 bias-corrected bootstrap resamples. Share of total indirect effect: 41% via M1, 59% via M2.

**Table 9:** Path estimates under conventional, cluster-robust and wild-cluster inference.

Path	*B*	SE (conventional)	SE (CR1)	*p* (CR1, df = 6)	*p* (wild-cluster bootstrap)
a₁: X → M1 (ERQ-R)	0.418	0.073	0.057	<0.001	0.006
a₂: X → M2 (MEIM-R)	0.425	0.050	0.045	<0.001	0.004
b₁: M1 → Y	0.234	0.039	0.049	0.003	0.010
b₂: M2 → Y	0.326	0.057	0.044	<0.001	0.004
c′: X → Y (direct)	0.161	0.068	0.073	0.071	0.090
c: X → Y (total)	0.397	0.066	0.078	0.002	0.017

7 institutions; CR1 = cluster-robust standard errors with a t distribution on 6 df; Webb wild-cluster bootstrap. Conventional standard errors are from the re-estimated models and differ slightly from Table 8.

Moderated mediation results appear in Table 10. Training intensity moderated the emotion-regulation pathway (index of moderated mediation = 0.006, [0.001, 0.012]). The conditional indirect effect increased from 0.061 at −1 SD to 0.123 at +1 SD of practice hours, tentatively supporting Hypothesis 7. The index was small and its interval close to zero, and the underlying X × W1 path to emotion regulation was only marginally significant (a₃ = 0.026, *p* = 0.048), so this moderation should be treated as tentative; under institution-level resampling the interval for the index touched zero (Table 11). Because the index excludes zero, the indirect effect is statistically moderated by practice hours; by contrast, the interaction on the direct tradition-wellbeing path (H6) was not significant. Cultural congruence did not significantly moderate the identity pathway (index = 0.025, [−0.004, 0.063]), although the conditional indirect effects were positive at every level of congruence (0.109–0.160). Hypothesis 5 was therefore not supported.

**Table 10:** Moderated mediation: conditional indirect effects and indices of moderated mediation.

Effect	Estimate	Boot SE	95% BC bootstrap CI	*p* (paths) / CI excludes 0
W1 (training intensity) → emotion-regulation pathway (M1)				
a₁ X → M (at mean W1)	0.423			<0.001
a₃ X × W1 → M	0.026			0.048
b₁ M → Y	0.217			<0.001
c′ direct X → Y	0.310			<0.001
Conditional indirect effect, W1 = −1 SD	0.061	0.024	[0.021, 0.117]	Yes
Conditional indirect effect, W1 = mean	0.092	0.023	[0.054, 0.143]	Yes
Conditional indirect effect, W1 = +1 SD	0.123	0.031	[0.070, 0.194]	Yes
Index of moderated mediation (a₃b₁)	0.006	0.003	[0.001, 0.012]	Yes
W2 (cultural congruence) → ethnic-cultural identity pathway (M2)				
a₁ X → M (at mean W2)	0.413			<0.001
a₃ X × W2 → M	0.076			0.116
b₁ M → Y	0.325			<0.001
c′ direct X → Y	0.257			<0.001
Conditional indirect effect, W2 = −1 SD	0.109	0.030	[0.058, 0.179]	Yes
Conditional indirect effect, W2 = mean	0.134	0.029	[0.083, 0.198]	Yes
Conditional indirect effect, W2 = +1 SD	0.160	0.037	[0.096, 0.241]	Yes
Index of moderated mediation (a₃b₁)	0.025	0.017	[−0.004, 0.063]	No

Bias-corrected bootstrap CIs (10,000 resamples). Conditional indirect effects evaluated at ±1 SD of the moderator.

**Table 11:** Indirect effects and indices of moderated mediation with participant-level and institution-level bootstrap intervals.

Effect	Estimate	95% CI (participant bootstrap)	95% CI (cluster bootstrap)	Cluster CI excludes 0
Indirect via M1 (a₁b₁)	0.098	[0.057, 0.146]	[0.066, 0.135]	Yes
Indirect via M2 (a₂b₂)	0.139	[0.086, 0.196]	[0.096, 0.185]	Yes
Total indirect	0.236	[0.172, 0.308]	[0.175, 0.297]	Yes
W1 pathway, conditional indirect, −1 SD	0.061	[0.017, 0.112]	[0.021, 0.108]	Yes
W1 pathway, conditional indirect, mean	0.092	[0.052, 0.139]	[0.053, 0.139]	Yes
W1 pathway, conditional indirect, +1 SD	0.123	[0.068, 0.189]	[0.067, 0.209]	Yes
W1 pathway, index of moderated mediation	0.006	[0.000, 0.012]	[−0.000, 0.013]	No
W2 pathway, conditional indirect, −1 SD	0.109	[0.054, 0.174]	[0.077, 0.139]	Yes
W2 pathway, conditional indirect, mean	0.134	[0.079, 0.195]	[0.095, 0.171]	Yes
W2 pathway, conditional indirect, +1 SD	0.160	[0.093, 0.235]	[0.104, 0.215]	Yes
W2 pathway, index of moderated mediation	0.025	[−0.005, 0.060]	[0.006, 0.050]	Yes*

W1 = training intensity (emotion-regulation pathway); W2 = cultural congruence (ethnic-cultural identity pathway). *Interval from resampling three or four institutions per tradition; unstable and not interpreted as support for H5.

The multilevel models (Table 12) showed that institutions accounted for 7.5% of the variance in wellbeing in the null model (ICC = 0.075). This fell to 0.007 once tradition was entered (γ₀₁ = 0.397, SE = 0.080) and to zero when practice hours and congruence were added. Within institutions, training intensity predicted wellbeing (0.023, *p* < 0.001). The multilevel estimate for tradition had a standard error (0.080, df = 5) about 20% larger than the HC3 estimate in Table 7 (0.066) but remained significant (*p* < 0.01). Because tradition is a level-2 variable that is fully confounded with institution and only seven institutions were sampled, the estimate should be read cautiously and cannot separate pedagogy from institution-level differences.

**Table 12:** Multilevel models of wellbeing (participants nested in institutions).

Parameter	Model 0: Null	Model 1: + Tradition	Model 2: + W1, W2
Intercept (γ₀₀)	0.024 (0.084)	−0.199*** (0.058)	−0.197*** (0.046)
Tradition (γ₀₁, level 2; df = 5)	—	0.397** (0.080)	0.393** (0.065)
Training intensity (level 1)	—	—	0.023*** (0.006)
Cultural congruence (level 1)	—	—	0.049 (0.033)
Institution intercept variance (τ₀₀)	0.0420	0.0036	0.0000
Residual variance (σ^2^)	0.5211	0.5204	0.5057
ICC (institution)	0.075	0.007	0.000
−2 log-likelihood (ML)	1062.4	1051.4	1034.9
AIC (ML)	1066.4	1057.4	1044.9

Random-intercept models, ML estimation. Tradition is constant within institution, so a random slope is not identified; with 7 institutions its test uses df = 5. **p < 0.01, ***p < 0.001.

In the longitudinal sub-sample (*n* = 192; Table 1), Western trainees’ wellbeing was stable (−0.20 to −0.17; time *B* = 0.004, *p* = 0.184), whereas Chinese ethnic trainees improved from 0.22 to 0.50. The time × tradition interaction was significant (*B* = 0.042, 95% CI [0.034, 0.050], *p* < 0.001). Depressive symptoms in the Chinese ethnic group declined from 5.3 to 3.9 while the Western group’s remained near 6.5 (interaction *B* = −0.276, *p* < 0.001). Latent growth and cross-lagged models are reported in the robustness section (Tables 13, 14). These models describe group differences in change; they do not test mediation or temporal precedence, they do not remove the confounding of tradition with institution and country, and no attrition analysis is reported here.

**Table 13:** Latent growth models of wellbeing and depressive symptoms (tradition predicting intercept and slope).

Outcome	Parameter	Estimate	SE	*p*
Wellbeing composite	Tradition → intercept	0.413	0.106	<0.001
Wellbeing composite	Tradition → slope (per 3 months)	0.124	0.015	<0.001
PHQ-9	Tradition → intercept	−1.109	0.485	0.022
PHQ-9	Tradition → slope (per 3 months)	−0.831	0.152	<0.001

Fit: wellbeing composite *χ*^2^(2) = 0.67, *p* = 0.715, CFI = 1.00, TLI = 1.00, RMSEA = 0.000; PHQ-9 *χ*^2^(2) = 1.45, *p* = 0.484, CFI = 1.00, TLI = 1.00, RMSEA = 0.000. Random-slope mixed models: time × tradition *B* = 0.126 (SE = 0.018, *p* < 0.001) for the composite and −0.828 (SE = 0.153, *p* < 0.001) for the PHQ-9; slope variances 0.006 and 0.612.

**Table 14:** Cross-lagged panel models (standardized coefficients).

Lag	Outcome	Autoregressive *β*	Cross-lagged *β*	Tradition *β*	*R* ^2^
T1 → T2	Wellbeing (T2)	0.949*	−0.019	0.143*	0.968
T1 → T2	PHQ-9 (T2)	0.894*	0.017	−0.177*	0.812
T2 → T3	Wellbeing (T3)	0.949*	−0.002	0.163*	0.961
T2 → T3	PHQ-9 (T3)	0.878*	−0.001	−0.277*	0.848

*p < 0.05. The wellbeing composite contains −z(PHQ-9), so these models are partly circular; see text.

Institutional means (Table 15) showed that the Yunnan, Guangxi, and Inner Mongolia programs had similar composites (0.26–0.29), whereas Beijing, with its mixed bel canto–minzu training, fell nearer the Western sites (−0.03 versus −0.25 to −0.13). In sum (Table 16), seven of the ten hypotheses were supported and Hypothesis 7 was tentatively supported (its index was borderline under cluster-based inference), while Hypotheses 5 and 6 were not supported. This pattern is descriptive. The Beijing program is also located in China, so it is not consistent with a simple country-level explanation, but with a single site it cannot isolate pedagogy from other institutional differences.

**Table 15:** Study measures by institution.

Institution	Tradition	*n*	Composite	Ryff	PHQ-9	STAI-T	ERQ-R (M1)	MEIM-R (M2)
New England Conservatory (Boston, USA)	Western	80	−0.21	145.9	6.53	46.0	4.64	3.37
Royal College of Music (London, UK)	Western	80	−0.25	142.1	6.15	46.4	4.67	3.29
Univ. of Music and Performing Arts (Vienna, Austria)	Western	80	−0.13	147.7	5.92	46.0	4.76	3.24
Central Conservatory of Music (Beijing)	Chinese ethnic	60	−0.03	150.4	5.88	45.6	5.13	3.66
Yunnan Arts University (Kunming)	Chinese ethnic	60	0.29	159.4	5.35	41.7	5.09	3.76
Guangxi Arts University (Nanning)	Chinese ethnic	60	0.26	160.8	5.35	43.3	4.99	3.68
Inner Mongolia Normal University (Hohhot)	Chinese ethnic	60	0.27	160.2	4.65	44.2	5.23	3.81

Descriptive means by institution.

**Table 16:** Summary of hypothesis tests (H1–H7).

Hypothesis	Statement	Key statistic	Decision
H1	Tradition is associated with mental wellbeing (total association c)	c = 0.397, *p* < 0.001	Supported
H2a	Tradition is associated with emotion regulation (a₁)	a₁ = 0.418, *p* < 0.001	Supported
H2b	Tradition is associated with ethnic-cultural identity (a₂)	a₂ = 0.425, *p* < 0.001	Supported
H3a	Emotion regulation is associated with wellbeing (b₁)	b₁ = 0.234, *p* < 0.001	Supported
H3b	Ethnic-cultural identity is associated with wellbeing (b₂)	b₂ = 0.326, *p* < 0.001	Supported
H4a	Indirect effect via emotion regulation	a₁b₁ = 0.098, CI [0.060, 0.147]	Supported
H4b	Indirect effect via ethnic-cultural identity	a₂b₂ = 0.139, CI [0.089, 0.201]	Supported
H5	Cultural congruence (W2) moderates identity pathway	IMM = 0.025, CI [−0.004, 0.063]	Not supported
H6	Intensity (W1) moderates direct tradition–wellbeing link	β₄ = 0.019, *p* = 0.108	Not supported
H7	Intensity (W1) moderates pathway via emotion regulation	IMM = 0.006, CI [0.001, 0.012]	Tentatively supported

Decisions use α = 0.05 (two-tailed) or a bootstrap CI that excludes zero.

### 4.1 Qualitative results

Codebook thematic analysis of the T3 interviews (*n* = 40) was organized around Braun and Clarke’s six phases. Two coders independently produced 32 initial codes, which were consolidated into four themes (Table 17). Inter-coder agreement was substantial across themes (κ = 0.76–0.84). The themes map onto the quantitative pathways, and their prevalence differed by tradition.

**Table 17:** Themes, frequency, initial coding and inter-coder agreement.

Theme	Example initial codes	Participants, *n* (%)	Chinese ethnic/Western	Coded segments	Initial codes (n)	Cohen’s *κ*
1. Breath as embodied calm	Slowing before performance; shoulders and breath; sustained phrasing; calming in daily stress	36 (90.0)	19/17	84	9	0.84
2. Belonging and ancestral continuity	Songs carry family and place; language pride; being held by ancestors; ensemble as family	31 (77.5)	20/11	71	8	0.81
3. Cultural congruence and teacher relationship	Teacher from the tradition; gentle correction; feeling understood; outsider teacher	27 (67.5)	16/11	52	7	0.78
4. Evaluative pressure and burden of intensity	Jury anxiety; ranking; overpractise; performing culture for a grade	29 (72.5)	11/18	63	8	0.76
Total / mean		40	20/20	270	32	0.80

Percentages use *n* = 40. κ = Cohen’s kappa on a double-coded subset of transcripts.

#### 4.1.1 Theme 1: breath as embodied calm

This was the most widely shared theme, appearing in 36 of 40 interviews. Participants in both traditions described controlled breathing as a practical way to settle the body before performance and in daily stress. Chinese ethnic vocalists more often linked breath to long phrases that had to be sustained together with a group, so calm was described as something practiced collectively rather than privately. Western participants more often described breath as an individual technical tool. These accounts describe participants’ subjective experience of calm; they do not index physiological regulation.

“When I sing urtiin duu the breath has to go on and on. If I panic, the line breaks. So I learned to let my shoulders drop first.” (P07, Chinese ethnic, Hohhot).

“Before a jury I do the same four slow breaths my teacher taught me. It doesn’t remove the fear, but my hands stop shaking.” (P31, Western, London).

#### 4.1.2 Theme 2: singing as belonging and ancestral continuity

This theme appeared in 31 interviews, but in every Chinese ethnic participant (20 of 20) compared with 11 of 20 Western participants. Chinese ethnic vocalists described songs as carrying family, place and language, and said that performing them affirmed who they were. Several described feeling “held” by earlier generations. Western participants who raised belonging usually spoke of their ensemble or studio rather than of heritage.

“These songs belong to my grandmother’s village. When I sing them I am not alone, I am part of a long line.” (P12, Chinese ethnic, Kunming).

“My choir is my family in this city. But it isn’t a history, it is more a friendship.” (P26, Western, Vienna).

#### 4.1.3 Theme 3: cultural congruence and the teacher relationship

Twenty-seven participants discussed how the teacher’s background and manner shaped their sense of safety. Participants valued teachers who came from, or were deeply rooted in, the tradition they taught, and described this as credibility that could not be replaced by technical skill. Congruence was often intertwined with the warmth and autonomy the teacher allowed, so participants rarely described it as a separate quality.

“My teacher is from the same grassland. He corrects me gently, and I trust that he understands what the song means.” (P17, Chinese ethnic, Hohhot).

“She is brilliant, but she has never lived with this music. I sometimes wonder whether she hears what I am trying to say.” (P04, Chinese ethnic, Beijing).

#### 4.1.4 Theme 4: evaluative pressure and the burden of intensity

Twenty-nine participants described pressure from juries, competition and heavy practice loads. This was more frequent among Western participants (18 of 20) than Chinese ethnic participants (11 of 20). Western participants tied anxiety to being ranked, whereas Chinese ethnic participants at the Beijing program, which blends bel canto and minzu training, described comparable strain. Higher practice hours were seen as beneficial when the singing felt communal and as exhausting when it was assessed. These accounts suggest a possible conditional or non-linear role for practice load, but this is a hypothesis for future testing: the quantitative tests of quadratic moderation did not support an inverted-U pattern (Tables 18, 19), and the interviews cannot establish a threshold or optimal number of weekly hours.

**Table 18:** Quadratic moderation tests (X × W^2^).

Moderator	Outcome	X × W^2^ B (SE)	*p*	W^2^ in Western group B (p)	Added-variance test	ΔAIC (linear − quadratic)
W1 training intensity	Wellbeing composite	0.0015 (0.0014)	0.308	−0.0014 (0.148)	*F*(2, 474) = 0.87, *p* = 0.420	−2.2
W1 training intensity	Mediator, a-path	−0.0019 (0.0017)	0.255	0.0006 (0.498)	*F*(2, 474) = 0.57, *p* = 0.567	−2.9
W2 cultural congruence	Wellbeing composite	−0.0265 (0.0565)	0.638	0.0475 (0.204)	*F*(2, 474) = 1.03, *p* = 0.357	−1.9
W2 cultural congruence	Mediator, a-path	0.0076 (0.0431)	0.860	−0.0346 (0.255)	*F*(2, 474) = 1.29, *p* = 0.276	−1.4

HC3 robust standard errors. Negative ΔAIC favors the linear model.

**Table 19:** Inverted-U check by group.

Moderator	Group	Quadratic term *B* (p)	Slope at observed minimum (p)	Slope at observed maximum (p)	Inverted *U* supported
W1 training intensity	Western	−0.0014 (0.148)	0.045 (0.044)	−0.032 (0.339)	No
W1 training intensity	Chinese ethnic	0.0001 (0.940)	0.030 (0.226)	0.035 (0.352)	No
W2 cultural congruence	Western	0.0475 (0.204)	−0.270 (0.206)	0.176 (0.239)	No
W2 cultural congruence	Chinese ethnic	0.0209 (0.621)	−0.020 (0.937)	0.176 (0.244)	No

Inverted *U* requires a significantly positive slope at the minimum and a significantly negative slope at the maximum of the moderator.

“Every semester there is a jury and everything is a number. I practice more, and I enjoy it less.” (P22, Western, Boston).

“In the village we just sang. Here we are graded, and some days I feel I am performing my culture for a score.” (P02, Chinese ethnic, Beijing).

Taken together, the interviews suggest that regulation and belonging are experienced as intertwined rather than separate, and that the training climate can strengthen or weaken both (Table 2). These themes provide context for, but cannot statistically explain, the quantitative estimates, including the residual direct effect of tradition.

### 4.2 Robustness and sensitivity analyses

Cluster-robust inference (Tables 9, 11). Re-estimating the path coefficients with CR1 standard errors for the seven institutions and Webb wild-cluster bootstrap *p*-values left a₁, a₂, b₁, b₂ and the total effect c significant (wild-cluster ps = 0.004–0.017), although *p*-values were larger than under conventional inference. The direct effect did not remain significant (c′ = 0.161; CR1 *p* = 0.071; wild-cluster *p* = 0.090, compared with *p* = 0.018 under conventional errors), so the earlier description of partial mediation is not robust to clustering; with seven clusters, power is limited, and the data are compatible with both a small direct association and none. All three indirect effects stayed clearly non-zero when whole institutions were resampled (via emotion regulation 0.098, cluster-bootstrap 95% CI [0.066, 0.135]; via ethnic-cultural identity 0.139, [0.096, 0.185]; total 0.236, [0.175, 0.297]). The index of moderated mediation for training intensity was borderline, its cluster-bootstrap interval touching zero ([−0.000, 0.013]), so Hypothesis 7 is treated as tentatively supported. The index for cultural congruence became significant under institution-level resampling ([0.006, 0.050]) but not under participant-level resampling ([−0.005, 0.060]); because resampling three or four institutions per tradition gives unstable and artificially narrow intervals, we do not treat this as support for Hypothesis 5, which remains unsupported.

Latent growth and cross-lagged models (Tables 13, 14). Latent growth models with tradition predicting intercept and slope fitted well (wellbeing composite χ^2^(2) = 0.67; PHQ-9 χ^2^(2) = 1.45; CFI ≈ 1.00; RMSEA = 0.000). The Chinese ethnic group had a steeper slope per 3 months for the wellbeing composite (0.124, SE = 0.015, *p* < 0.001) and for the PHQ-9 (−0.831, SE = 0.152, *p* < 0.001), and random-slope mixed models gave the same results. Slope variance was very small (0.006 for the composite), so the models mainly capture group differences in average change rather than individual differences in trajectories. Cross-lagged paths between wellbeing and PHQ-9 were near zero (|β| ≤ 0.02) and autoregressive paths were high (0.88–0.95), so there was no evidence of lagged reciprocal prediction over three-month intervals. This test is partly circular because the wellbeing composite contains −z(PHQ-9), and stability this high leaves little variance for cross-lagged paths to explain. None of these longitudinal models tests mediation or removes the confounding of tradition with institution and country.

Curvilinear moderation (Tables 18, 19). Adding quadratic moderation terms did not improve the models. The X × W^2^ terms were not significant for training intensity (*p* = 0.308 on wellbeing; *p* = 0.255 on the regulation path) or for cultural congruence (*p* = 0.638; *p* = 0.860), the added-variance tests against the linear models were non-significant (ps = 0.276–0.567), and AIC favored the linear models in every case. In no group was the slope significantly positive at the observed minimum and significantly negative at the observed maximum of the moderator, so an inverted-U or “sweet spot” pattern was not supported. The only significant slope was positive in the Western group at low practice hours (0.045, *p* = 0.044), which does not match an inverted U. Interview accounts that long practice hours can be helpful or exhausting therefore remain a hypothesis that the quantitative tests did not support.

## 5 Discussion, implications, and limitations

The results are consistent with the pathways proposed in the VP-PWIM but cannot establish them. Chinese ethnic vocal training was associated with higher psychological wellbeing (H1), and this association was statistically accompanied by indirect associations through self-reported emotion regulation and ethnic-cultural identity (H2-H4). Because tradition is completely confounded with institution, region and country, and because the mediation models are cross-sectional, these patterns cannot be attributed to pedagogy itself and the indirect effects should not be read as causal mechanisms. The indirect associations were robust to cluster-based inference, whereas the direct association was not, so the claim of partial mediation should be treated as unsupported. The pattern agrees with evidence that culturally embedded musical participation is linked with mental health across community and educational settings (Chen et al., 2025; Huang et al., 2025; Sigwebela et al., 2026). The ethnic-cultural identity pathway was the larger of the two, and tradition differences were greatest for ethnic-cultural identity. This fits Phinney’s (1990) and Phinney and Ong’s (2007) argument that a secure sense of belonging to a valued group supports wellbeing, the meta-analytic link between ethnic identity and wellbeing (Smith and Silva, 2011), and social identity theory (Tajfel and Turner, 1979). However, the MEIM-R referent and its meaning may differ between a Chinese group studying minority repertoire and an internationally diverse Western group, and invariance was not tested, so the large group difference on this measure should be read with that in mind. Interview accounts of songs as carriers of family, language and place offer one possible interpretation of why repertoire tied to specific communities may be linked to stronger identity in ways generic technical training may not; this interpretation needs testing.

The emotion-regulation pathway should be interpreted narrowly. The ERQ-R captures self-reported cognitive reappraisal; it does not measure vagal tone, parasympathetic activation, respiration or any other physiological process, so the pathway cannot serve as evidence for polyvagal mechanisms. The association is compatible with the psychophysiological literature on slow, controlled breathing (Zaccaro et al., 2018) and with polyvagal accounts of how vocalization and social engagement support calm (Porges, 2001, 2003, 2007), and singing has been shown to synchronize heart rate variability among singers (Vickhoff et al., 2013) and group singing to promote rapid social bonding (Kreutz et al., 2004; Pearce et al., 2015; Weinstein et al., 2016). Testing such an account would require physiological indicators such as heart-rate variability, respiratory sinus arrhythmia or respiratory measures. The interviews echoed these accounts at the level of subjective experience, since participants described sustaining long phrases together as a form of shared calm. Training intensity was associated with a stronger regulation pathway (H7), but the index was small, its interval touched zero under institution-level resampling, and the moderation of the direct effect (H6) was not significant. Some interviewees described long practice hours as helpful when singing felt communal and exhausting when it was assessed; this points to a possible conditional role for practice load, but formal tests of quadratic moderation found no support for an inverted-U or “sweet spot” pattern (Tables 18, 19), so the idea remains a hypothesis for future studies and is not a result of this one.

Two hypotheses were not supported, and both deserve interpretation. Cultural congruence did not significantly moderate the identity pathway (H5). Self-determination theory suggests why: congruence may operate together with autonomy support and relatedness rather than independently (Ryan and Deci, 2000; Evans, 2015), and interview participants rarely separated cultural credibility from a teacher’s warmth. Controlling or need-thwarting relationships are known to diminish functioning (Bartholomew et al., 2011), so a congruence scale that ignores relational climate may underestimate its effect. The lower anxiety in the Chinese ethnic group also sits against a background of well-documented performance anxiety in conservatory and vocal students (Kenny, 2005; Okan and Usta, 2021; Racine et al., 2024; Steptoe and Fidler, 1987; Ye, 2026; Zhukov et al., 2026). The evaluative pressure described in Theme 4 offers one possible interpretation of this strain, namely that assessment climate rather than singing itself contributes to it; the interviews cannot statistically explain the residual direct effect of tradition, and this interpretation remains to be tested.

These findings carry theoretical and practical implications. Theoretically, they illustrate how self-determination, polyvagal and social identity perspectives can be combined into one testable model in which regulation and identity are candidate complementary routes to wellbeing. Practically, conservatories could include minority repertoire, recruit or train teachers who are culturally grounded, and adopt autonomy-supportive feedback and less punitive jury formats. Music learning has been linked to psychological development through self-efficacy and self-esteem (Bi and Rauduvaitė, 2025; Jiang, 2024; Lewis and Hendricks, 2022), and pedagogy that values the acoustic and stylistic distinctiveness of traditional Chinese singing (Liu and Wallmark, 2024; Pan, 2023) may help students realize these benefits without abandoning technical rigor. These suggestions are provisional, because the present design cannot show that adopting such practices would improve student wellbeing.

Several limitations qualify these conclusions. First, the design is observational and comparative, not quasi-experimental. Students were not randomly assigned to traditions, and tradition is completely confounded with institution, curriculum, region, country, recruitment context, socioeconomic and cultural environment, and institutional prestige. With only seven institutions the level-2 test has limited power, and the multilevel model cannot separate tradition from institution. Differences in anxiety, depression and wellbeing may therefore reflect broader national or institutional differences, such as academic pressure structures, rather than pedagogy. Future work should sample both traditions within the same setting, for example Western bel canto and Chinese ethnic vocal majors within one Chinese conservatory, using crossed or cross-classified designs and, where feasible, randomized or waitlist designs. Second, the mediation and moderation models are cross-sectional and cannot establish temporal order or causal mechanisms; the longitudinal sub-sample was modest, was analyzed only for group differences in change, and is subject to attrition. Latent growth models showed steeper improvement in the Chinese ethnic group, but they do not test mediation, and the cross-lagged models are partly circular because the composite contains the PHQ-9 and showed near-zero cross-lagged paths with very high stability. Third, all measures were self-reported. The PHQ-9 (Kroenke et al., 2001) screens for symptoms rather than diagnosing depression, and the ERQ-R captures reappraisal (Gross and John, 2003) but not other strategies; no physiological indices were collected, so the polyvagal mechanism was not tested. Fourth, measurement invariance across the Western and Chinese groups was not tested, translated instruments were used in one group, and the MEIM-R may not carry equivalent meaning in both groups; the wellbeing composite is an equal-weight mean of standardized scores and was not validated as a latent construct. Fifth, the primary mediation estimates use participant-level inference. Cluster-robust re-estimation with only seven institutions made the direct effect non-significant and left the training-intensity moderation borderline, and with so few clusters even the cluster-robust estimates are imprecise. Sixth, the sample comes from institutional programs, participants’ own ethnic backgrounds are not equated with the traditions they study, and the results should not be generalized to community-based Indigenous or other community knowledge holders, or to ethnic traditions beyond those sampled (Yi, Bai, Dai, Zhuang and Mongolian programs). Finally, the qualitative strand used a codebook approach with a limited number of interviews per site, and its themes offer context and plausible interpretation rather than statistical explanation.

## 6 Conclusion

In this observational comparison across seven institutions, students in Chinese ethnic minority vocal programs reported higher wellbeing and ethnic-cultural identity, and lower anxiety and depressive symptoms, than students in Western conservatory programs, and these differences were statistically accompanied by indirect associations through self-reported emotion regulation and ethnic-cultural identity. Because tradition is completely confounded with institution and country, the mediation models are cross-sectional, and the emotion-regulation measure does not index physiological processes, the findings do not show that Chinese ethnic vocal pedagogy produces better wellbeing or that polyvagal mechanisms are involved. Interviews described breath, belonging, teacher congruence and evaluative pressure as salient features of training and gave context for the numerical patterns. The study is best read as hypothesis-generating. Stronger tests would sample both traditions within the same settings, use longitudinal and physiological designs, and measure identity with instruments validated across groups. Even so, the results suggest that culturally grounded repertoire and autonomy-supportive, lower-pressure assessment climates merit further evaluation as supports for student wellbeing in conservatories.

## Data Availability

The original contributions presented in the study are included in the article/supplementary material, further inquiries can be directed to the corresponding author/s.
